# Distal gut colonization by oral bacteria during intensive chemotherapy: direct evidence from strain-level analysis of paired samples

**DOI:** 10.1038/s41522-025-00725-7

**Published:** 2025-05-26

**Authors:** Suravi Raychaudhuri, Hakan Gem, Kevin Chung, Jeffrey S. McLean, Kristopher A. Kerns, Meredith A. J. Hullar, Elissa Elmorr, Jacob B. Appelbaum, Mary-Elizabeth M. Percival, Roland B. Walter, Anna B. Halpern, Samuel S. Minot, Katie Kim, Alexander S. Zevin, Armin Rashidi

**Affiliations:** 1 Clinical Research Division, Fred Hutchinson Cancer Center, Seattle, WA USA; 2 Division of Hematology and Medical Oncology, Department of Medicine, University of Washington, Seattle, WA USA; 3 Department of Oral Medicine, University of Washington, Seattle, WA USA; 4 School of Dentistry, University of Washington, Seattle, WA USA; 5 Clinical Oral Microbiome Research Center, University of Washington, Seattle, WA USA; 6 Public Health Sciences Division, Fred Hutchinson Cancer Center, Seattle, WA USA; 7 Translational Science and Therapeutics Division, Fred Hutchinson Cancer Center, Seattle, WA USA; 8 Data Core, Shared Resources, Fred Hutchinson Cancer Center, Seattle, WA USA; 9 Genomics and Bioinformatics Shared Resource, Fred Hutchinson Cancer Center, Seattle, WA USA

**Keywords:** Metagenomics, Health care

## Abstract

Oral bacteria have been found in the colon in pathologies such as inflammatory bowel disease. To ascertain niche coalescence, 2 elements are essential: (i) paired oral/fecal samples and (ii) strain-level resolution. We profiled the microbiota in 283 samples from 39 patients undergoing intensive chemotherapy at baseline (saliva: 49, plaque: 51, stool: 43), week 2 (saliva: 18, plaque: 17, stool: 17), week 3 (saliva: 18, plaque: 21, stool: 21), and week 4 (saliva: 8, plaque: 10, stool: 10) of chemotherapy. Through strain-level analysis of paired samples, we demonstrate strong evidence for a breakdown of niche separation in most patients. The extent of overlap increased with time, particularly in patients with intestinal mucositis. Our findings provide definitive evidence for ectopic colonization of the distal gut by oral bacteria in a disease state, likely facilitated by intestinal mucositis. Microbiota contribution by the mouth to the colon may have consequences for the host.

## Introduction

The mouth and colon are distinct ecological niches for the microbiota, with nearly no overlap in healthy adults^1–3^, although some controversy exists^4^. Despite swallowing 1-1.5 liters of saliva containing 10^11^ oral bacteria per day^5^, physiological barriers along the gastrointestinal tract (e.g., stomach acid, intestinal bile, antimicrobial peptides) limit the survivability of passenger microbes. In addition, the vastly different physicochemical properties (e.g., oxygen pressure) of the mouth and colon select for uniquely adapted microbial communities in the two habitats. Therefore, even the small fraction of oral microbes that may reach the colon alive do not survive there, let alone actively contribute to the colonic microbial ecology^6^. In addition, colonization resistance mediated by the distal gut microbiota opposes the survival, expansion, and activity of the incoming microbes via the saliva, although the relative importance of this mechanism has been debated^7^.

Oral bacteria might be able to ectopically colonize the distal gut in pathological states, leading to niche coalescence. Oral inflammation in mice exacerbates colitis; the mechanism involves expansion of proinflammatory oral pathobionts that translocate to the gut^8,9^. In humans, colonization of the distal gut with bacterial species of likely oral origin has been associated with inflammatory bowel disease^10–12^. However, whether ectopic colonization is the cause or a consequence of mucosal inflammation is unknown. Murine data support a colitogenic effect for specific oral pathobionts^8^. On the other hand, intestinal mucosal inflammation and epithelial injury (e.g., due to cytotoxic chemotherapy or immune attack) can increase oxygen levels in the normally hypoxic colonic lumen, promoting the survival and expansion of aerobic/facultatively anaerobic oral microbes^13,14^.

Intensive chemotherapy for blood cancers represents a unique opportunity to understand the occurrence, extent, and significance of ectopic colonization of the distal gut by oral microbiota in humans, as well as to dissect its cause-and-effect relationship with intestinal mucositis. These patients experience major disruptions to their oral and colonic microbiota and frequently develop intestinal mucositis due to direct cytotoxic effects from chemotherapy. Previous work using short-amplicon sequencing to profile the microbiota and operational taxonomic units (OTU) for classification suggested a major coalescence of oral and colonic microbiota in these patients^15^, while re-examination of data using short-amplicon exact sequence variants found only a minute level of overlap^7^. The level of resolution in taxonomic identification is critical in this question because true overlap requires ascertainment of the presence of the same bacteria at the strain level in the two samples. Indeed, the proinflammatory effect of oral pathobionts in the colon seems to be strain-specific^10^. However, due to the limited within-species genetic variation within a given hypervariable region of the 16S rRNA gene, short-amplicon methods generally do not provide the required level of resolution beyond genus^16^.

To unequivocally demonstrate niche coalescence and thereby invoke evidence for ectopic colonization, strain-level data from paired samples (i.e., samples from the oral and colonic microbiota taken at the same time and from the same individual) are needed. We generated a shotgun metagenomic database of longitudinal multi-site microbiota from paired salivary, supragingival plaque, and stool samples from patients with blood cancers undergoing intensive chemotherapy. Using strain-level analysis, we provide definitive evidence for ectopic colonization of the distal gut by oral bacteria in these patients. In addition, we find that intestinal mucositis can facilitate this process.

## Results

### Patients, clinical events, and samples

Data from 39 patients were analyzed (Table 1). The median age was 60 years (range 31–78) and 22 (56%) were male. Antibiotic exposures are summarized as density plots in Fig. 2a, indicating peak exposure times for each antibiotic class. As expected, exposure to antibiotics used to treat neutropenic fever or infections (e.g., 3rd or higher generation cephalosporins, vancomycin, and anti-anaerobic antibiotics) peaked at the end of week 2 and beginning of week 3—during the period of neutropenic nadir and therefore of most profound immunosuppression. In contrast, no peak in exposure was apparent for prophylactic fluoroquinolones used throughout the entire period of neutropenia. All patients started treatment in the hospital, as is the standard of care for intensive chemotherapy. The median length of the first hospitalization was 12 days (range 5–42). Twenty-one (54%) patients were readmitted following discharge from the hospital, all due to infection/fever. Two (5%) patients died by day 30: 1 due to infection and 1 due to stress cardiomyopathy. A median of 2 discrete fever/infection episodes (range 0–6) occurred per patient by day 30. The two most common types of these events were neutropenic fever without a microbiologically documented infection (41% of events) and bloodstream infection (20% of events). Severe intestinal mucositis occurred in 12 (31%) patients by day 30. Clinically significant oral mucositis did not occur in any patient.

**Table 1:** Patient characteristics

Total	39
Sex, *N* (%)	
Male	22 (56%)
Female	17 (44%)
Age, median (range)	60 (31-78) years
Disease, *N* (%)	
Acute myeloid leukemia	33 (85%)
Other high-grade myeloid malignancy	6 (15%)
Disease type, *N* (%)	
De novo	32 (82%)
Treatment-related	4 (10%)
Secondary	3 (8%)
Disease phase, *N* (%)	
New diagnosis	33 (85%)
Relapsed/Refractory	6 (15%)
Chemotherapy regimen, *N* (%)	
CLAG-M + /− GO +/− midostaurin	35 (90%)
FLAG-Ida + venetoclax	3 (7%)
Liposomal daunorubicin and cytarabine (Vyxeos)	1 (3%)
First hospitalization length, median (range)	12 (5-42) days
Fever/Infection episodes by day 30, *N* (%)^a^	
Median (range)	2 (0-6)
Number of episodes	10 (28%)
1	14 (36%)
2	9 (23%)
3	2 (5%)
4	2 (5%)
6	1 (3%)
Fever/Infection type, *N* (%)^b,c^	
Neutropenic fever without microbiological documentation	37 (41%)
Bloodstream infection	18 (20%)
Skin and soft tissue infection	9 (10%)
Lower respiratory tract infection	9 (10%)
Gastrointestinal infection	8 (9%)
Urogenital infection	3 (3%)
Oral cavity infection	3 (3%)
Upper respiratory tract infection	2 (2%)
Readmission by day 30 of chemotherapy, *N* (%)^d^	21 (54%)
Death by day 30 of chemotherapy, *N* (%)	2 (5%)
Infection	1
Stress cardiomyopathy	1

*CLAG-M*: cladribine + cytarabine + G-CSF + mitoxantrone; *FLAG-Ida*: fludarabine + cytarabine + G-CSF + idarubicin; GO: gemtuzumab ozogamicin.^a^Episodes at least 24 h apart were considered 2 episodes. ^b^When fever and infection occurred as the same episode, the episode was considered an infection and fever was not considered separately. ^c^Percentages are relative to the total number of episodes (*N* = 89). ^d^The cause of all readmissions was infection/fever.

**Fig. 1:**
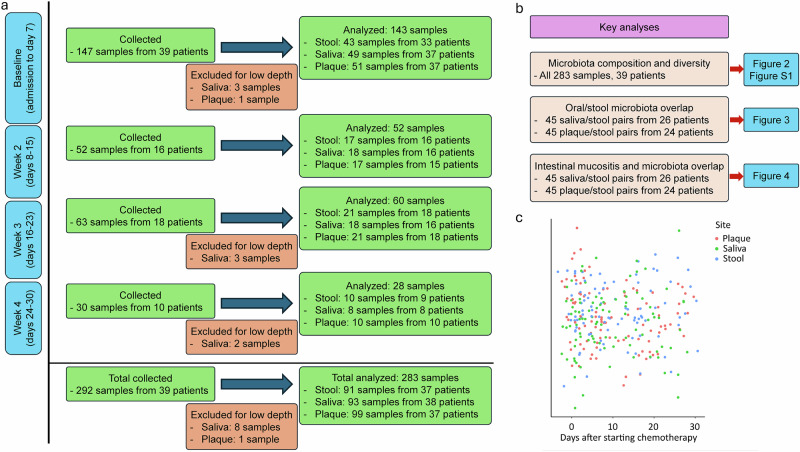
Study design, samples, and patients. **a** Flowchart of samples and patients at each timepoints. **b** Material used in key analyses. **c** Sample distributions over time. The y axis does not represent a variable, but allows for a jitter to avoid sample superimposition.

A total of 292 samples were sequenced, 9 (3.0%; bl saliva=3, w3 saliva=3, w4 saliva=2, bl plaque=1) of which were removed due to low depth, resulting in 283 samples (saliva: 93; plaque: 99; stool: 91) for the final analysis. These samples were collected at bl (saliva: 49, plaque: 51, stool: 43), w2 (saliva: 18, plaque: 17, stool: 17), w3 (saliva: 18, plaque: 21, stool: 21), and w4 (saliva: 8, plaque: 10, stool: 10). The median interval between 2 consecutive samples of the same type from the same patient was 7.5 days (interquartile range 7–10). A detailed flowchart of samples and patients is shown in Fig. 1. The median number of high-quality microbial reads per sample was 18.5 M.

### Microbiota composition changes and its diversity declines during chemotherapy

**Fig. 2:**
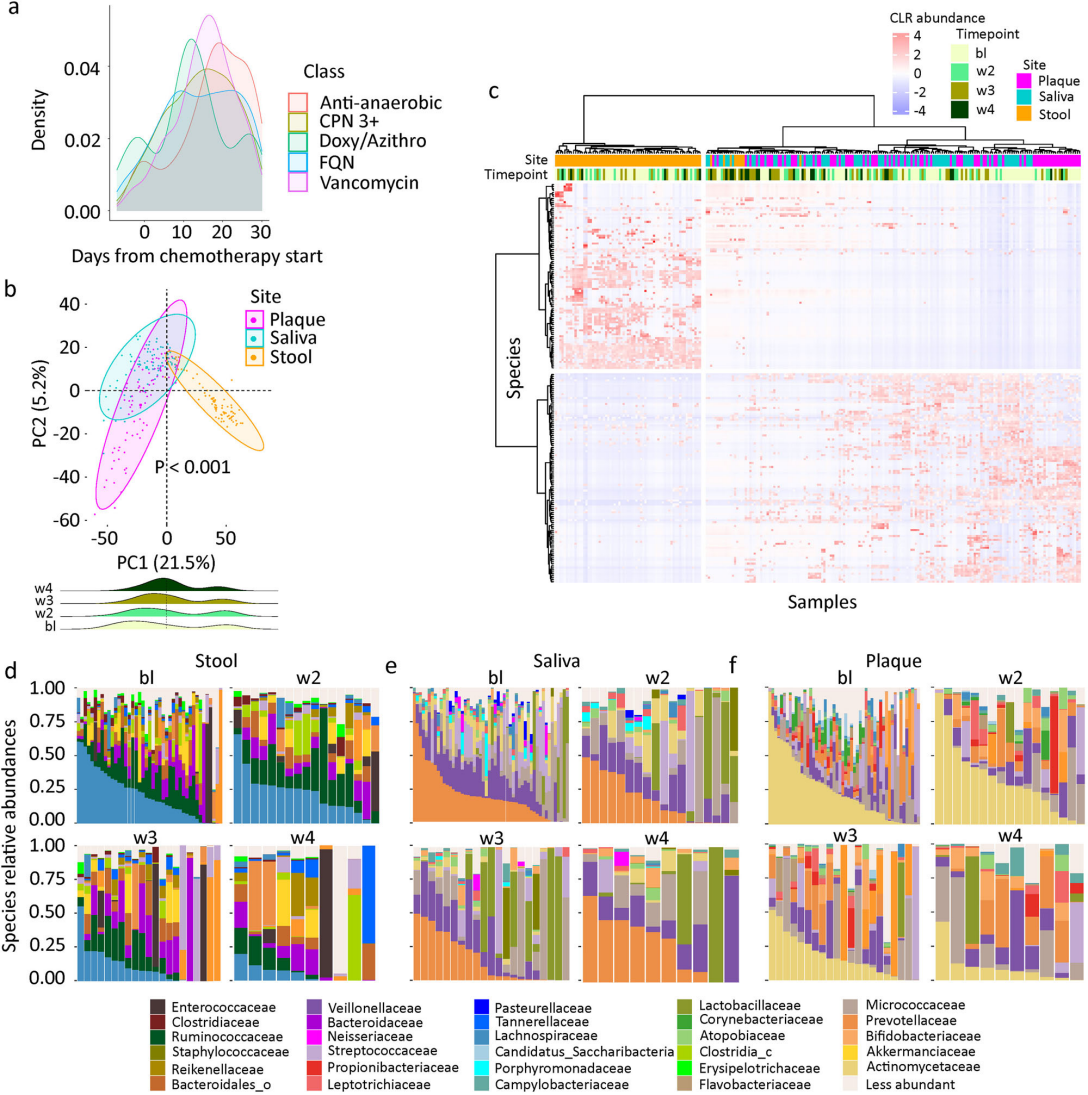
Microbiota composition. **a** Kernel density plot of antibiotic exposures. The 5 most common antibacterial antibiotic exposures are included. Azithro: azithromycin; CPN 3 + : 3rd or higher generation cephalosporins; Doxy: doxycycline; FQN: fluoroquinolones. **b** Beta diversity and ordination visualized by principal coordinate analysis. Aitchison’s distance was used to quantify the overall compositional difference between samples. The first two principal coordinates (PC1 and PC2) are shown. Numbers in parentheses indicate percent variation explained by the corresponding axis. Each symbol represents a sample. The centroid of each cluster is shown by a larger symbol. 95% ellipses are shown. A timepoint density plot is added at the bottom border. **c** Species-level microbiota heatmap visualizing the results of unsupervised hierarchical clustering using centered log-ratio transformed abundances and a ward.D function. Each column is a sample and each row is a species. The blue-red gradient shows species abundances scaled row-wise. Timepoints and sites are added along the top border. **d–f** Family-level microbiota composition grouped based on site and timepoint, with the 15 most abundant families in each site shown. Less abundant families are grouped together. c: class; o: order (insufficient resolution for family classification).

First, we examined general patterns in microbiota composition and diversity. As previous studies showed a decrease in alpha diversity of both oral and stool microbiota and changes in their composition (e.g., reduction of *Blautia* and *Faecalibacterium* and expansion of *Lacticaseibacillus* in the gut microbiota) during chemotherapy^15,17–19^, we investigated whether we could reproduce similar findings. In principal coordinates analysis, the most prominent determinant of microbiota variation was sample site, explaining 18.7% of the variation (*P* < 0.001), and consistent with previous analyses^15,20^. This was followed by timepoint, explaining 2.0% of microbiota variation (*P* = 0.008). Adjustment for batch did not change these results (sample site: 18.8%, *P* < 0.001; timepoint: 2.1%, *P* = 0.002), arguing against a batch effect. Samples of each site clustered separately from those of the other sites (overall and pairwise PERMANOVA *P* < 0.001, adonis test with 999 permutations; Fig. 2b), indicating site specificity of microbiota composition. In species-level hierarchical clustering (Fig. 2c), and as expected, stool samples were the most distinct. As shown in Fig. 2d–f, the 3 most abundant families in the stool were Lachnospiraceae, Ruminococcaceae, and Bacteroidaceae. For saliva, these were Prevotellaceae, Veillonellaceae, and Micrococcaceae. For plaque, these were Actinomycetaceae, Micrococcaceae, and Veillonellaceae. These findings are consistent with the expected distinct microbiota compositions of saliva, plaque, and stool, supporting the internal validity of our methodology.

To further evaluate microbiota dynamics, we compared microbiota composition at each non-baseline timepoint to baseline, using patient ID as a random effect in MaAsLin2 (Supplementary data 1). The most significant changes in the microbiota in all 3 sites occurred between bl and w3/w4. For plaque, these changes included a relative expansion of *Lacticaseibacillus rhamnosus* and *Scardovia wiggsiae*, and reduction of *Streptococcus oralis* and *Corynebacterium matruchotii*. For stool, major changes included a relative expansion of *L. rhamnosus* and reduction of several *Blautia* species (*B. wexlerae*, *B. obeum*, and *B. massiliensis*) and *Faecalibacterium prausnitzii*. For saliva, major changes included a relative expansion of *S. wiggsiae* and *L. rhamnosus*, and reduction of *Gemella sanguinis*, *Haemophilus parainfluenzae*, and *Streptococcus parasanguinis*. Collectively, *L. rhamnosus* expansion was a common feature of microbiota changes over time in all 3 sites.

Alpha diversity dynamics in the 3 sites are shown in Supplementary Fig. S1. To analyze the effect of site and time on alpha diversity while accounting for data longitudinality, we built a linear mixed model with Shannon diversity as the outcome variable, site, time (days relative to chemotherapy initiation), and site x time interaction as fixed effects, and patient ID as a random effect. As the interaction term was not significant (i.e., similar diversity decline rate across sites; *P* = 0.17 for stool vs. plaque microbiota and *P* = 0.19 for saliva vs. plaque microbiota), it was removed and a simpler model without interaction was built. In this analysis (Table 2), both sample site and time were significant predictors of microbiota diversity. Specifically, stool had the highest diversity, while plaque and saliva had a similar but lower diversity. Diversity declined over time and at a similar rate in all 3 sites.

**Table 2:** Mixed model for alpha diversity dynamics

	Regression coefficient	95%CI	*P*
Time	−0.04	−0.05 to −0.03	<0.001
Sample site (vs. plaque)			
Saliva	−0.11	−0.30 to 0.08	0.27
Stool	0.49	0.29 to 0.69	<0.001

Fixed effects were time (days relative to chemotherapy initiation) and sample site (relative to plaque). Patient ID was a random effect. Shannon diversity was the outcome variable. 95%CI: 95% confidence interval.

### A significant fraction of the gut microbiota has an oral origin

So far, our findings demonstrated a significant decline in microbiota diversity and compositional changes in all sites over time. The principal coordinates analysis and density plots in Fig. 2b suggest some degree of niche coalescence at later timepoints as previously suggested^15^, hence the possibility of ectopic colonization of the distal gut by oral microbiota. To demonstrate this with certainty, we proceeded with within-patient strain-level analysis using paired oral/stool samples. While finding the same strain in paired oral and stool samples from the same patient would be strongly supportive of ectopic colonization, we also considered a much less likely explanation, namely, a relative expansion of an already overlapping strain to detectable levels. The latter would require (*i*) pre-existing overlap and (*ii*) higher levels of ecological resistance and adaptability than other less-adaptable bacteria in the face of perturbations in microbiota-host homeostasis during chemotherapy.

**Fig. 3:**
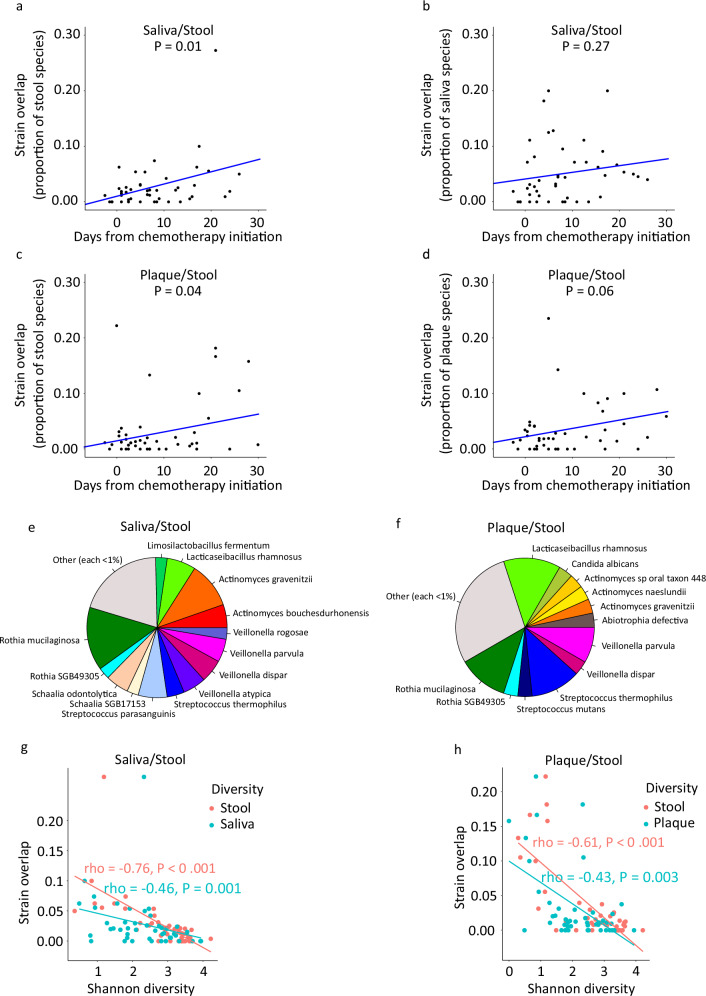
Oral/gut microbiota overlap. Strain-level overlap between saliva and stool microbiota over time in within-patient paired saliva/stool samples and measured as the fraction of species in the stool (**a**) or saliva (**b**) sample that was overlapping as the same strain. The *P* value is from a mixed model with overlap as the outcome variable, time as a fixed effect, and patient ID as a random effect. **c**, **d** Same analysis as in panels (**a**, **b**), but for plaque instead of saliva samples. **e** Fraction of saliva/stool overlapping strains belonging to different species. **f** Fraction of plaque/stool overlapping strains belonging to different species. **g** Correlation between strain-level saliva/stool microbiota overlap and alpha diversity of each site. Correlation coefficients (rho) and *P* values are from a Spearman’s correlation test. **h** Same analysis as in panel (**g**), but for plaque instead of saliva samples. To generate panels (**a**, **b**, **g**), 45 sample pairs from 26 patients (median of 2 sample pairs/patient) were used. To generate panels (**c**, **d**, **h**), 45 sample pairs from 24 patients (median of 2 sample pairs/patient) were used.

To distinguish between these possibilities, we first identified saliva/stool sample pairs collected on the same day from the same patient. Forty-five such sample pairs from 26 patients were available, with a median of 2 sample pairs per patient (range 1–4). There was an overlap of at least 1 strain in 35 (78%) sample pairs from 23 (88%) patients. Among all sample pairs, the overlapping strains constituted a median of 1.9% (range 0–27.3%) and 4.0% (range 0–20%) of the species in the stool and saliva, respectively. The overlap proportion increased with time for stool (*P* = 0.01), but not saliva (*P* = 0.27) in mixed models with overlap as response variable, time as a fixed effect, and patient ID as a random effect (Fig. 3a-b). The same analysis was repeated for plaque/stool sample pairs. Forty-five sample pairs from 24 patients were available, with a median of 2 sample pairs per patient (range 1–4). There was an overlap of at least 1 strain in 33 (73%) sample pairs from 23 (96%) patients. Among all sample pairs, the overlapping strains constituted a median of 1.1% (range 0–22.2%) and 1.9% (range 0–23.5%) of the species in the stool and plaque, respectively. These proportions increased with time (*P* = 0.04 and 0.06, respectively; mixed model with overlap as response variable, time as a fixed effect, and patient ID as a random effect; Fig. 3c, d). The species most frequently containing the overlapping strains between saliva and stool were *Rothia mucilaginosa*, followed by *Actinomyces graevenitzii*, *S. parasanguinis*, and *L. rhamnosus* (Fig. 3e). The species most frequently containing the overlapping strains between plaque and stool were *L. rhamnosus*, followed by *Streptococcus thermophilus*, *R. mucilaginosa*, and *Veillonella parvula* (Fig. 3f). Intriguingly, *Candida albicans* was the only fungus with overlapping strains between oral (plaque) and stool samples. The extent of oral/stool microbiota overlap strongly and negatively correlated with the diversity of both oral and stool microbiota, with less diverse pairs exhibiting more overlap (Fig. 3g, h).

Overall, *R. mucilaginosa* and *L. rhamnosus* contained the largest fraction of overlapping strains. In site-specific differential abundance analysis (see above), *L. rhamnosus* expanded in all sites over time; however, neither *R. mucilaginosa* nor any of the other species containing site-overlapping strains expanded over time; there was even significant reduction of one species (*A. graevenitzii*) with overlapping strains in the saliva over time. The earliest that same-strain *L. rhamnosus* overlap was identified in any oral/stool sample pair was at day 7. None of the 76 overlap events (i.e., same-strain overlap in an oral/stool sample pair) before day 7 was due to this species. These observations argue against a model where the overlapping strains were highly resistant to ecological perturbations, adaptable, and pre-existing in both niches and reached detectable levels of relative abundance merely due to a preferential elimination of less adaptable bacteria during chemotherapy. *R. mucilaginosa*, the other species with overlapping strains in many cases, is an oral commensal and its same-strain co-existence in the oral/gut microbiota is most consistent with translocation from the mouth to the colon. Together, the best model supported from these findings is one of ectopic colonization and a significant strain-level contribution of oral microbiota to distal gut microbiota in most patients, especially at later times when microbiota diversity declines.

To examine taxonomic differences between sample pairs collected from patients with vs. without coalescence (in at least 1 strain in at least 1 sample pair), we first performed a PERMANOVA test (adonis with 999 permutations) for each sample site (saliva, plaque, and stool) separately. Time (i.e., days from chemotherapy initiation) and group (patients with vs. without coalescence) were included in each model. For all 3 sample sites, both time (as we saw earlier) and group were significant contributors to microbiota variation (Supplementary Table S1). Next, we used MaAsLin2 to identify species that were differentially abundant between the two groups. In this analysis, time and group were the potential predictors. Supplementary data 2 lists species-level taxonomic differences between the groups. As opposed to the decline of several species (in all 3 sample sites) in the group with coalescence, only a few species expanded: *Bacteroides fragilis* in the stool, *L. rhamnosus* and *S. wiggsiae* in the saliva, and *L. rhamnosus* and *R. mucilaginosa* in the plaque. The most remarkable of these changes were the last two (*P* values 0.017 and 0.015; regression coefficients 4.6 and 4.2, respectively). Although *L. rhamnosus* expanded in both saliva and plaque in patients experiencing coalescence, the expansion in the saliva (*P* = 0.04, coefficient 3.0) was much less prominent than in the plaque. As *L. rhamnosus* and *R. mucilaginosa* contained the largest fraction of overlapping strains, these findings suggest that plaque was the main contributor of microbiota to the distal gut. Expansion of specific taxa in the plaque possibly facilitated their translocation to the colon. These taxa did not reach high relative abundances in the colon, likely due to the higher abundance of gut-native microbiota.

### Intestinal mucositis may increase ectopic colonization of the oral microbiota

**Fig. 4:**
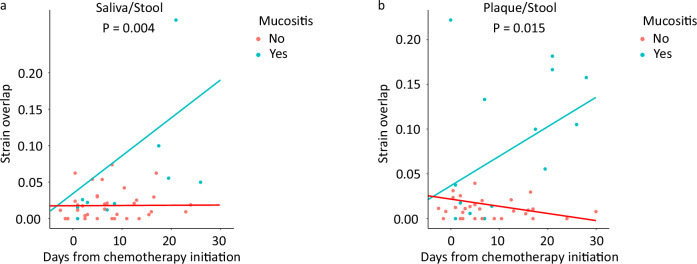
Possible effect of intestinal mucositis on niche overlap. **a** Change in strain-level saliva/stool microbiota overlap over time in patients with vs. without severe intestinal mucositis. The *P* value corresponds to the regression coefficient for the time x mucositis interaction term in a mixed model with overlap as the outcome variable, time, mucositis, and time x mucositis as fixed effects, and patient ID as a random effect. Mucositis refers to severe intestinal mucositis. *P* < 0.05 indicates different slopes for the two lines, i.e. significant effect of mucositis on the dynamics of overlap. **b** Same analysis as in panel (**a**), but for plaque instead of salivary microbiota. To generate panel (**a**), 45 within-patient stool/saliva sample pairs from 26 patients (median of 2 sample pairs/patient) were used. To generate panel (**b**), 45 within-patient stool/plaque sample pairs from 24 patients (median of 2 sample pairs/patient) were used.

Next, we investigated whether intestinal mucositis might facilitate colonization of the distal gut by oral bacteria. In this exploratory analysis, using 45 within-patient stool/saliva sample pairs from 26 patients (median of 2 sample pairs/patient), the fraction of gut microbiota that coexisted as the same strain in the saliva increased over time in patients with severe intestinal mucositis but remained unchanged in those without severe intestinal mucositis (*P* = 0.004 for time x mucositis interaction in a mixed model; Table 3, Fig. 4a). A similar pattern was found in stool/plaque analysis using 45 within-patient stool/plaque sample pairs from 24 patients (median of 2 sample pairs/patient) (*P* = 0.015 for time x mucositis interaction in a mixed model; Table 3, Fig. 4b).

**Table 3:** Mixed models for oral/gut microbiota overlap dynamics

Saliva/stool microbiota overlap			
	Regression coefficient	95%CI	*P*
Time	0.0002	−0.0017 to 0.0021	0.86
Mucositis (yes vs. no)	-0.0147	−0.0553 to 0.0259	0.46
Time x mucositis	0.0051	0.0019 to 0.0083	0.004

Plaque/stool microbiota overlap			
	Regression coefficient	95%CI	*P*
Time	−0.0001	−0.002 to 0.002	0.91
Mucositis (yes vs. no)	0.023	−0.016 to 0.062	0.23
Time x mucositis	0.0036	0.0008 to 0.006	0.015

In each mode, time (days from chemotherapy initiation), severe mucositis (yes vs. no), and time x mucositis interaction were fixed effects. Patient ID was a random effect. Mucositis refers to severe intestinal mucositis. Strain-level overlap was the outcome variable. 95%CI: 95% confidence interval.

### Clinical predictors and significance of niche coalescence

Finally, we evaluated whether patients who experienced niche coalescence had different baseline characteristics or experienced different clinical outcomes compared to patients without coalescence. To this end, we started with the set of 30 patients who had at least 1 oral/stool sample pair and classified them into two groups: (*i*) with coalescence in at least one sample pair (*n* = 23), (*ii*) without coalescence in any sample pair (*n* = 7). Because the unit of analysis here was patients rather than samples, our sample size substantially decreased, resulting in insufficient power for any definitive statistical comparison. Thus, the results of this analysis should be interpreted with caution.

The two groups were relatively similar in age (*P* = 0.25), sex (*P* = 1.00), hospitalization length (*P* = 0.26), and disease phase (*P* = 0.07). However, the proportion of patients with acute leukemia was higher in the group with coalescence (22 of 23; 96%) vs. without coalescence (4 of 7; 57%). Antibiotic exposures were different between the groups (Supplementary Fig. S2a-b). The group with coalescence experienced heavier exposure to 3rd or higher generation cephalosporins, antianaerobic antibiotics, vancomycin, and doxycycline/azithromycin, while the group without coalescence experienced heavier exposure to fluoroquinolones. As fluoroquinolones were used exclusively prophylactically in these patients, the latter finding suggests fewer fever/infection episodes in the group without coalescence, consistent with the statistical trend observed in the analysis of fever/infection (see below). In addition, peak exposure to most antibiotic classes was about 1 week later in the group without vs. with coalescence (Supplementary Fig. S2c-d).

There was a trend for more infection/fever episodes by day 30 in the group with coalescence (*P* = 0.15), with a median of 2.5 (range, 1–6) events vs. 2 (range, 0–3) in the group without coalescence. Of the 5 patients with coalescence who proceeded to allogeneic hematopoietic cell transplantation, 2 (40%) developed grade II-IV acute graft-versus-host disease (1 isolated skin; 1 lower gut). Of the 7 patients in the group without coalescence who proceeded to transplantation, 1 (14%) developed grade II-IV acute graft-versus-host disease (1 isolated skin).

## Discussion

Strain-level analysis of longitudinal, multi-site, within-individual paired samples allowed us to address whether in a clinical setting with numerous ecological perturbations at the host-microbiota interface, the two arguably most distinct microbiota niches in the healthy state – oral and distal gut microbiota - overlap. Our two main novel findings from this analysis are: (*i*) Niche coalescence occurs in most patients undergoing intensive chemotherapy, with oral microbiota comprising >5% of the colonic microbiota in many patients; (*ii*) Chemotherapy-induced intestinal mucositis seems to facilitate this process.

The two species containing the largest fraction of overlapping strains were *R. mucilaginosa* and *L. rhamnosus*. *R. mucilaginosa* is a Gram-positive facultatively anaerobic commensal species found abundantly in the oral cavity and upper respiratory tract^21–23^, and only rarely in other body sites. Therefore, strain-level coexistence of this species in paired samples indicates an oral origin, i.e., ectopic colonization of the gut by oral microbiota. *L. rhamnosus* is a Gram-positive facultatively anaerobic commensal species also marketed as a probiotic, with a large genome and negligible niche specialization. It is found in different body sites of both humans and other vertebrates as well as in food products^24^. Adaptations to both gut and oral environments allow this species to exist in both niches, at least for short periods^25^. These adaptations include metabolic flexibility enabling fermentation of a wide array of carbohydrates^26^, bile acid resistance^27^, mucus binding ability^28^, and immunomodulation (e.g., NF-κB response via Toll-like receptor 2)^29^. Importantly, while the low pH in the stomach is a major barrier against bacterial translocation through the stomach, *L. rhamnosus* can survive this harsh environment through glucose-mediated proton exclusion^30–32^. The complete absence of this species on the list of species with overlapping strains in oral/stool sample pairs before day 7 of chemotherapy supports ectopic colonization of the gut by oral-origin *L. rhamnosus* rather than prior presence of this species in both niches followed by its relative expansion during chemotherapy. Both *R. mucilaginosa* and *L. rhamnosus* expanded in the plaque (but not stool) more significantly in patients who experienced niche coalescence compared to those who did not. This finding further supports ectopic colonization of the gut by plaque microbiota. *Candida albicans* was the only fungus in our analysis with overlapping strains between paired oral (plaque) and stool samples. Cross-feeding and interkingdom symbiotic relationships between this fungus and *Streptococcus mutans* contribute to their plaque biofilm formation^33,34^. Candida colonization of the colon is common in healthy adults but can lead to translocation and candidemia in immunocompromised patients receiving gut toxic chemotherapy^35^.

We are aware of only one previous study examining oral/gut microbiota overlap in patients undergoing intensive chemotherapy^15^. The study collected longitudinal saliva and stool samples from patients with acute myeloid leukemia undergoing intensive chemotherapy. Analysis was performed using short-amplicon 16S rRNA gene OTUs. The degree of coalescence increased with time and correlated negatively with alpha diversity, consistent with our findings. Patients who experienced coalescence had a higher incidence of infections than those without coalescence, again consistent with the trend seen in our analysis. The potential effect of intestinal mucositis on coalescence was not explored. As the authors mentioned, OTU-based analysis limited the confidence in establishing true coalescence. We re-analyzed the same dataset, but using short amplicon sequence variants (ASVs) with single-nucleotide resolution. Although we found a much lower degree of overlap between oral and stool samples than in the reported findings from OTU-based analysis, one of the few overlapping ASVs belonged to the genus *Lacticaseibacillus*. Although species assignment could not be unambiguously performed due to short amplicon sequencing, this observation is consistent with our results in the present work. Another OTU-based analysis, this time in critically ill children, also suggested coalescence of tongue dorsum/stool microbiota^36^. We advanced these studies by implementing shotgun sequencing combined with strain-level analysis of concurrently collected sample pairs, demonstrating definitive evidence for substantial oral/gut microbiota coalescence in most sample pairs. Over 75% of patients experienced coalescence, making this a common phenomenon among patients undergoing intensive chemotherapy.

Although the presence of bacteria with typical or presumed oral origin in the gut or even species-level overlap in paired samples, as has been best demonstrated in inflammatory bowel disease^37^, may reflect a true overlap, it cannot be considered definitive because different strains of the same species may be adapted to very different ecological niches. An elegant study of oral and fecal microbiota from patients with Crohn’s disease combined with mechanistic, acute and chronic murine models of inflammatory bowel disease showed that periodontitis may promote gut colonization of salivary *Haemophilus parainfluenzae* in a strain-specific manner, eliciting intestinal inflammation^10^. In another study of paired saliva/stool samples from healthy adults and patients with colorectal cancer, rheumatoid arthritis, or type 1 diabetes, microbial single nucleotide variants (SNV) in metagenomic sequences were used to define strains^4^. SNVs were then used to derive a transmission score per subject, quantifying how much the similarity between oral and gut SNV profiles within an individual deviated from an inter-individual background. The authors found evidence for substantial niche overlap in healthy individuals and patients with colorectal cancer or rheumatoid arthritis. Overlapping strains belonged to *Streptococcus*, *Veillonella*, *Actinomyces*, and *Haemophilus*, among other core members of the oral microbiota.

Our study has some limitations. First, although strain-level analysis is the most precise sequencing-based method to examine niche overlap, it does not inform on transcriptionally active or even live bacteria. In a previous analysis of saliva/stool microbiota overlap analysis in healthy adults, the exceedingly rare overlapping bacteria were not transcriptionally active in the gut^6^. Whether our findings in a specific disease setting represent the same functionally neutral (albeit much more substantial) overlap as in healthy adults needs further study. Expansion of some of our overlapping stains over time supports the presence of live bacteria. Second, although the temporal sequence of events – high levels of overlap after, but not before intestinal mucositis – is strongly suggestive of causality, it does not prove it. Definitive demonstration of causality requires controlled experiments in animal models. Third, we cannot ascertain the pathway through which oral bacteria translocated to the colon. While saliva seems the most readily available means for translocation of plaque microbiota shed from the biofilm (destabilized due to chemotherapy and antibiotic damage), translocation from the oral cavity to the local submucosal lymph nodes and then to the gut is an alternative pathway, as shown in a previous murine study^8^. Finally, while patients with coalescence experienced heavier and earlier exposure to broad-spectrum antibiotics, especially those with strong anti-anaerobic activity, the relative contribution of these exposures to niche overlap (e.g., due to loss of gut microbiota-mediated colonization resistance) is unknown and requires further research.

In conclusion, we provide definitive evidence for strain-level oral/gut microbiota overlap in patients undergoing intensive chemotherapy, likely resulting from colonization of the distal gut by specific oral bacteria and facilitated by chemotherapy-induced intestinal mucositis. This ectopic colonization may worsen intestinal inflammation initiated by cytotoxic chemotherapy.

## Methods

We conducted a prospective, single-center, observational study including longitudinal stool and multi-site oral samples in adults (age ≥18) with high-grade myeloid malignancies receiving frontline or salvage intensive chemotherapy. No other inclusion or exclusion criteria were used. No statistical power calculation was performed, and enrollment was based on feasibility. The protocol was approved by the Fred Hutchinson Cancer Center’s Institutional Review Board. All patients provided written informed consent. Severe intestinal mucositis was defined as either grade 3+ diarrhea per the Common Terminology Criteria for Adverse Events (CTCAE) v.5.0 criteria or CT scan-based findings of enterocolitis.

### Sample collection

Saliva, supragingival plaque, and stool samples were collected approximately once a week starting as soon as possible after admission to the hospital and continued until discharge from the hospital or day 30 following chemotherapy initiation, whichever occurred first. While the timing of stool samples was inherently unplanned, we attempted to collect the oral samples as closely as possible to the stool samples. In general, chemotherapy was started on the day of or shortly after admission to the hospital. In some patients, however, this was delayed due to additional work up or specific clinical circumstances. Day 0 was defined as the first day of chemotherapy. For patients discharged and readmitted before day 30, collections were resumed. Four collection intervals were considered: (*i*) baseline (bl): admission to the hospital through day 7, (*ii*) week 2 (w2): day 8 through day 15, (*iii*) week 3 (w3): day 16 through day 23, and (*iv*) week 4 (w4): day 24 and later.

Saliva and plaque samples were collected at the same time (plaque immediately following saliva) and after at least 30 min of no oral intake or oral hygiene. Up to 5 mL of saliva was collected by passive drooling into a sterile tube containing 5 mL of sterile 95% ethanol. The tube was pulse vortexed for 5 s to ensure homogenous mixture of saliva and ethanol before immediate storage at −80 °C. Plaque samples were taken from 3 teeth (1 posterior and 2 anterior) using a sterilized scaler. Plaque samples were transferred from the scaler to the tip of a sterilized plastic pick. The pick was then submerged into a sterile tube containing 500 µL of sterile 95% ethanol. The pick was agitated until the clump of plaque fell off the instrument into ethanol. The tube was immediately transferred to −80 °C. Stool was self-collected from the toilet paper using a sterile swab, placed in a sterile tube containing 3 mL of sterile 95% ethanol, and kept at 4 °C until transfer to −80 °C. All stool samples were transferred to −80 °C within 5 days of collection. The use of 95% ethanol for storing oral and stool samples has been validated and allows for DNA preservation at room temperature for several weeks^38–40^.

### Microbiota sequencing

DNA was extracted using the QIAmp DNA Microbiome Kit (Qiagen, Germany) following the manufacturer’s protocol that uses both mechanical and chemical cell lysis. Extraction of stool and oral samples was performed in two different labs to minimize the risk of cross-contamination. Sample purification and quality control were performed as previously described^41^. Sequencing libraries were prepared using the Illumina^®^ DNA Library Preparation Kit (Illumina, San Diego, CA) following the manufacturer’s protocol and with unique dual-index 10 bp barcodes with Nextera^®^ adapters. All libraries were pooled in equal abundance and the final pool was quantified using qPCR and TapeStation^®^ (Agilent Technologies, Santa Clara, CA). The final library was sequenced on an Illumina NovaSeq 6000 using a S2-300 flow cell and a PE150 configuration. The ZymoBIOMICS^®^ Microbial Community DNA Standard (Zymo Research, Irvine, CA) was used as a positive control for each library preparation. Multiple negative controls (i.e. blank extraction control, blank library preparation control) were included to assess the level of bioburden carried by the wet-lab process. Other negative controls included sterile 95% ethanol with and without a blank swab.

Raw paired-end metagenomic sequence reads were quality-processed using the integrated pipeline provided in KneadData v.0.12.0. This sequence-level procedure included two main steps: (*i*) removal of reads mapped to the human reference genome GRCh37 (hg19) using Bowtie2 v.2.4.5^42^ and (*ii*) removal of adapter sequences and low-quality reads using Trimmomatic v.0.39^43^ with default settings. Output files consisting of surviving paired and orphan reads were concatenated and used as input to MetaPhlAn4^44^. MetaPhlAn4 with default parameters was used for species-level taxonomic assignment. MetaPhlAn4 uses a set of species-level genome bins (SGBs)^45^ as primary taxonomic units and accurately profiles their presence and abundance in metagenomes. The latest version (version 4) of MetaPhlAn uses a database containing ~5.1 million unique species-specific marker genes for 21,978 existing SGBs and 4992 yet-to-be-characterized SGBs (defined solely based on metagenome-assembled genomes). Samples with fewer than 500,000 high-quality microbial reads were excluded.

### Statistical analysis

All analyses were performed in R 4.2.0. When more than one sample from the same site and at the same timepoint were available from the same patient, they were pooled. This procedure was performed only when time was analyzed as categorical intervals (bl, w2, w3, and w4). The possibility of a batch effect was examined using *MMUPHin*^46^, with adjustment for site (saliva vs. plaque vs. stool) and timepoint. Within-sample (alpha) and between-sample (beta) diversity were determined from species-level data in vegan v.2.6.4 by Shannon’s index^47^ and Aitchison’s distance^48^, respectively. Ordination was visualized in principal coordinates analysis (i.e., principal components analysis applied to Aitchison distances) using *factoextra* v.1.0.7. Differential abundance analysis comparing the baseline microbiota to the microbiota at each non-baseline timepoint was performed by Microbiome Multivariable Association with Linear Models using MaAsLin2^49^. Species with ≥0.01 relative abundance in ≥10% of the samples were included. Patient ID was considered a random effect. Volcano plots were generated using P values and regression coefficients corresponding to species. To analyze the effect of site and time on alpha diversity while accounting for data longitudinality, we built a linear mixed model with Shannon diversity as the outcome variable, site, time (days from chemotherapy initiation), and site x time interaction as fixed effects, and patient ID as a random effect. This analysis was performed using the *nlme* package, with the MLE-based inference method to derive *P* values and 95% confidence intervals. No imputation for missing data was performed in longitudinal analysis; rather, subjects with unavailable samples at the specific timepoints included in each longitudinal analysis were omitted from analysis.

The overlap between oral and colonic microbiota was determined at the strain level using SameStr^50^ which leverages MetaPhlAn’s species-specific markers to resolve within-species phylogenetic sequence variations. First, MetaPhlAn marker alignments were converted to single nucleotide variant profiles. These profiles were then filtered, merged, and compared between samples based on the maximum variant profile similarity (MVS) to detect strains that were shared between samples. Shared strains were called if species alignments between samples overlapped by ≥5 kb and with an MVS of ≥99.9%. For each stool sample with a paired oral sample (saliva or plaque in separate analyses) collected on the same day, we determined the proportion of species in the stool sample that co-existed as the same strain in the paired oral sample. Co-existing species with insufficient resolution for strain-level analysis were not included in this calculation.

### Reporting summary

Further information on research design is available in the Nature Research Reporting Summary linked to this article.

## Supplementary information

Supplementary material

nr-editorial-policy-checklist

Reporting summary

Supplementary Data 1

Supplementary Data 2

## Data Availability

The sequencing data reported in this paper will be available from NCBI Sequence Read Archive (SRA) under a BioProject ID PRJNA1265886.
