# Supplementary material for: Distal gut colonization by oral bacteria during intensive chemotherapy: direct evidence from strain-level analysis of paired samples
Source: NPJ Biofilms Microbiomes. 2025 May 26;11:88. doi: 10.1038/s41522-025-00725-7 (PMC12106833; doi:10.1038/s41522-025-00725-7)
Supplement: Supplementary file 1 — Supplementary material [file 41522_2025_725_MOESM1_ESM.pdf]

Supplemental Information

Supplementary Table S1: Contribution of time and niche coalescence to microbiota variation

Stool		
	R^2	P
Time	0.02	0.012
Coalescence (yes vs. no)	0.02	0.008
Saliva		
	R^2	P
Time	0.05	0.009
Coalescence (yes vs. no)	0.02	0.001
Plaque		
	R^2	P
Time	0.04	0.012
Coalescence (yes vs. no)	0.02	0.001

The results are from PERMANOVA, using an adonis test with 999 permutations. Coalescence was defined binarily: 1 if the sample belonged to a patient who experienced strain-level overlap in at least 1 sample pair; 0 if the sample belonged to a patient who, despite having at least 1 sample pair, did not experience strain-level overlap in any sample pairs. Time was defined as days from chemotherapy initiation.

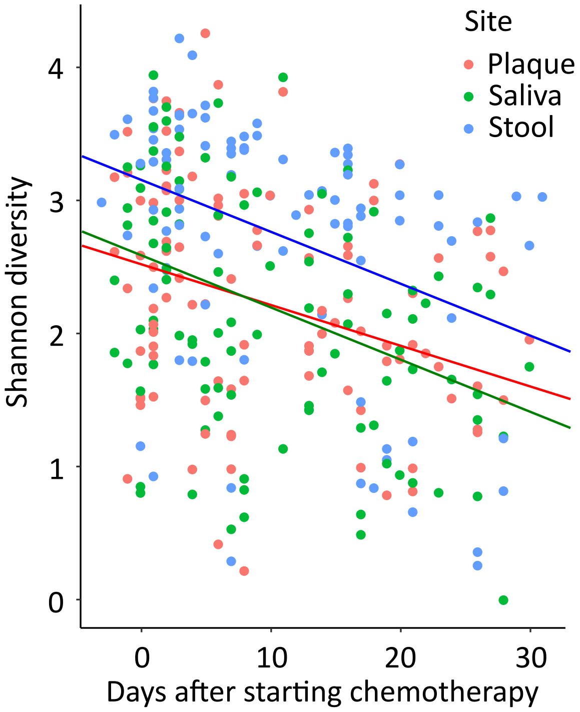

Supplementary Figure S1: Microbiota diversity over time

Alpha diversity as measured by the Shannon's index is shown. The intercept and slope of each regression line come from a mixed model, with Shannon diversity as the outcome variable, time (days from chemotherapy initiation) as a fixed effect, and patient ID as a random effect.

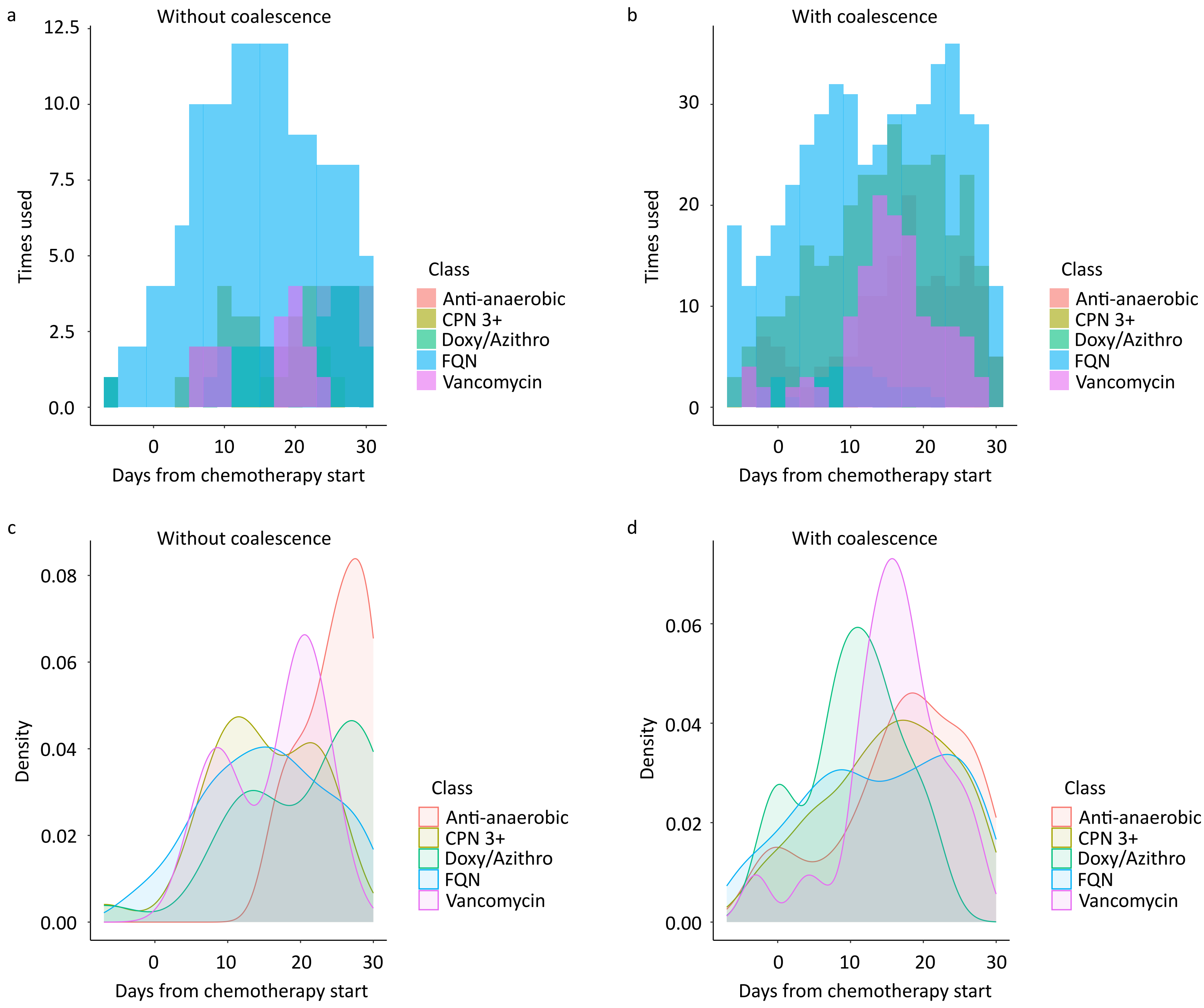

Supplementary Figure S2: Antibiotic exposures in patients with vs. without niche coalescence (a-b) Histogram of antibiotic exposures. (c-d) Kernel density plot of antibiotic exposures. The 5 most common antibacterial antibiotic exposures are included. Patients with coalescence are those with at least 1 oral/stool sample pair with at least 1 strain overlap. Patients without coalescence are those with at least 1 oral/stool sample pair but without any strain overlap in any such samples. Azithro: azithromycin; CPN 3+: 3rd or higher generation cephalosporins; Doxy: doxycycline; FQN: fluoroquinolones

Supplementary data 1: Species-level changes from baseline to subsequent timepoints
Each panel shows a comparison between a timepoint to baseline and for a specific site. P values are from MaAsLin2.

Supplementary data 2: Species-level differences between patients with vs. without oral/stool coalescence.
Patients with coalescence are those with at least 1 oral/stool sample pair with at least 1 strain overlap. Patients without coalescence are those with at least 1 oral/stool sample pair but without any strain overlap in any such pairs. P values are from MaAsLin2.
